# High-dose single-fraction IMRT versus fractionated external beam radiotherapy for patients with spinal bone metastases: study protocol for a randomized controlled trial

**DOI:** 10.1186/s13063-015-0761-7

**Published:** 2015-06-09

**Authors:** Harald Rief, Sonja Katayama, Thomas Bruckner, Stefan Rieken, Tilman Bostel, Robert Förster, Ingmar Schlampp, Robert Wolf, Jürgen Debus, Florian Sterzing

**Affiliations:** Department of Radiation Oncology, University Hospital of Heidelberg, Im Neuenheimer Feld 400, 69120 Heidelberg, Germany; Department of Medical Biometry, University Hospital of Heidelberg, Im Neuenheimer Feld 305, 69120 Heidelberg, Germany; Heidelberg Institute of Radiation Oncology, Im Neuenheimer Feld 280, 69120 Heidelberg, Germany

**Keywords:** Bone metastases, Spine, SBRT, IMRT, Palliative radiotherapy

## Abstract

**Background:**

Stereotactic body radiation therapy (SBRT)using intensity-modulated radiotherapy (IMRT) can be a safe modality for treating spinal bone metastasis with enhanced targeting accuracy and an effective method for achieving good tumor control and a rigorous pain response.

**Methods/design:**

This is a single-center, prospective randomized controlled trial to evaluate pain relief after RT and consists of two treatment groups with 30 patients in each group. One group will receive single-fraction intensity-modulated RT with 1×24 Gy, and the other will receive fractionated RT with 10×3 Gy. The target parameters will be measured at baseline and at 3 and 6 months after RT.

**Discussion:**

The aim of this study is to evaluate pain relief after RT in patients with spinal bone metastases by means of two different techniques: stereotactic body radiation therapy and fractionated RT. The primary endpoint is pain relief at the 3-month time-point after RT. Secondly, quality of life, fatigue, overall and bone survival, and local control will be assessed.

**Trial registration:**

ClinicalTrials.gov identifier NCT02358720 (June 2, 2015).

## Background

The vertebral column is the main localization of bone metastases and is where they frequently indicate an advanced stage of a malignant primary disease [1, 2]. Two thirds of all patients with tumors are estimated to develop bone metastases in the course of their disease [3]. Spinal bone metastases are of central impact for patients in a palliative setting. The clinical symptoms include pain at rest and under exercise but also impaired activity of daily life, the risk of pathological fractures, and neurological deficits. Pain is the essential factor for decreased quality of life (QoL) of patients with bone metastases. In regard to pain therapy and re-calcification of former osteolytic lesions, palliative radiotherapy (RT) represents an effective treatment option [4]. The most common schedule was specified as 30 Gy in 10 fractions. The outcome showed a partial pain response in 50 % to 80 % of patients and a complete pain response in one third [5]. Stereotactic body radiation therapy (SBRT) using intensity-modulated radiotherapy (IMRT) can be a safe modality for treating spinal metastasis with enhanced targeting accuracy [6]. Secondly, IMRT to the spine was well tolerated (especially in the spinal cord), had no significant late toxicities, and spared other organs at risk simultaneously [7]. Pretreatment megavoltage computed tomography (CT) allows clinicians to position control and correction to determine the localization of the metastasis, and to hold a divergence of the dose of the local region most minimally [8]. Nguyen *et al*. [9] showed that SBRT with 24 Gy is a safe and effective treatment modality that can be used to achieve good tumor control and palliation of pain associated with spinal metastases. To the best of our knowledge, no comparable randomized study has been described in the literature so far.

The aims of this study were to apply a high biological dose in the tumor region and to achieve a comparable result related to pain relief and local control on the one hand and to reduce the overall treatment time for palliative patients with painful spinal bone metastases on the other hand. Secondly, the aim was to evaluate QoL, fatigue, and survival.

## Methods/design

**Fig. 1:**
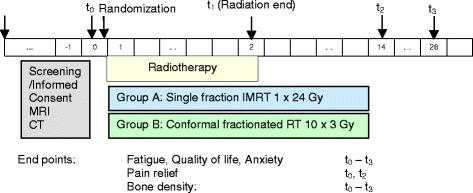
Flowchart of the study. CT, computed tomography; IMRT, intensity-modulated radiotherapy; MRI, magnetic resonance imaging; RT, radiotherapy

This is a single-center, prospective randomized controlled trial with parallel-group design to determine the pain relief after RT in patients with spinal bone metastases. Two different techniques were evaluated: high-dose single-fraction IMRT with 1×24 Gy and fractionated external beam RT with 10×3 Gy. Prior to enrolment in the study, the patients will undergo a staging of the vertebral column in connection with their CT for RT planning and magnetic resonance imaging (MRI) to measure the spinal cord dimension. After the baseline results have been recorded, the patients will be randomly assigned to one of the two groups: single-fraction IMRT 1×24 Gy (n = 30) or fractionated RT 10×3 Gy (n = 30). The target parameters will be measured and recorded at baseline, at the end of RT (t_1_), and at 12 weeks (t_2_) and 6 months (t_3_) after the end of the irradiation period (Fig. 1).

### Recruitment and randomization

The patients will be given information on the study by the medical personnel of the RT department in connection with the planning of the RT schedule (about 1 to 2 weeks prior to the start of RT). If they are interested in participating in the study, the study candidates will be given the patient information sheet, including the declaration of informed consent, with the request that they re-read the information carefully and, if they consent to the conditions, return the signed declaration at the next appointment. Patients will be given the opportunity to ask the study staff further questions. Informed consent will be obtained from each participant prior to enrolment in the study. Among the preconditions for participation in the study is the condition that no metastatic spinal cord compression (tumor distance of more than 3 mm to spinal cord) of the metastasized vertebral body be detected in the MRI recorded during the planning procedure.

A block randomization procedure shall be used to ensure the even distribution of patients into two intervention groups, stratified at baseline by pain level. The patients shall then be assigned 1:1 to one of the two treatment groups on the basis of the baseline measurements. The randomization procedure shall be carried out by a central office. The study personnel responsible for the recruitment and baseline measurements shall have no access to the randomization list. The recruitment phase shall be concluded with the attainment of the planned number of patients (60 patients in total). It shall last 12 months and was scheduled to start in December 2014. Regular study participation shall end 6 months after enrolment in the study or, where applicable, with the respective patient’s death.

### Inclusion criteria

- Patients with a histologically confirmed tumor diagnosis and with secondary diagnosed solitary/multiple spinal bone metastases
- Indication for RT of the spinal bone metastases
- Maximum of two irradiated vertebral bodies per region
- Maximum of two different vertebral regions
- Age of between 18 and 80 years
- Karnofsky index [10] of at least 70
- Signed declaration of informed consent
- Tumor distance of more than 3 mm to spinal cord.
- Exclusion criteria
- Multiple myeloma or lymphoma
- Significant neurological or psychiatric disorders, including dementia and epileptic seizures
- Contraindication for MRI
- Previous RT at the current irradiation site
- Other severe disorders that in the judgment of the study director may prevent the patient’s participation in the study
- Lacking or diminished legal capacity
- Any medical of psychological condition that the study director considers a preventive factor for the patient’s ability to complete the study or to adequately understand the scope of the study and to give his or her consent.

### Assessment of the primary and secondary endpoints

The aim of the trial is to evaluate pain relief after RT in patients with spinal bone metastases. Two different techniques are evaluated: single-fraction IMRT with 1×24 Gy and fractionated external beam RT with 10×3 Gy. The primary endpoint is defined as pain relief of more than 2 points according to visual analog scale (VAS) measured at the irradiated region at the 3-month time-point after the end of RT (t_2_). Secondary endpoints are QoL, fatigue, pain, overall survival, bone survival, local control, pathological fracture, and neurological deficit. The baseline examination shall be carried out on the first day of RT prior to the start of therapy and is scheduled to comprise the comprehensive recording of the socio-demographic data, the recording of the current pain situation, the fear of suffering fractures, the QoL, and the current degree of fatigue. The follow-up examinations shall take place after the end of RT (day of the last fraction) and 12 weeks and 6 months after RT, measuring those parameters recorded at the baseline examination. The further follow-up examinations shall correspond to those carried out as standard after-care investigations.

The secondary endpoints such as fatigue, QoL, and anxiety shall be recorded by using validated questionnaires (EORTC QLQ FA13 [11], EORTC QLQ BM22 [12], and the questionnaire to record stress in patients with cancer (FBK) according to Book *et al*. [13]). All patients will also be asked to record their pain history by using a pain diary (documentation of medication daily during treatment, once weekly after the end of treatment, VAS pain scale). Furthermore, the local control is assessed by means of CT images taken prior to and 3 and 6 months after RT. The pain response is documented on the VAS (range of 0 to 10). Complete response is defined as VAS score of 0 after 3 and 6 months, and partial response is defined as a score improvement by at least 2 points after 3 and 6 months, according to the international consensus response categories of Chow *et al*. [14]. Overall survival is defined as time from initial diagnosis until death, and bone survival is defined as time from initial diagnosis of spinal bone metastasis until death.

### Radiotherapy

Treatment is simulated with a CT simulator by using a 3-mm slice thickness taken within the involved vertebral region. Immobilization is ensured with an Aquaplast head mask (Aquaplast Corporation, Wyckoff, NJ, USA), vacuum mattress, and Wingstep® (Elekta, Stockholm, Sweden). On the basis of the planning CT, risk organs and clinical target volume (CTV) are contoured. The spinal cord is contoured on the basis of visible target on the CT scan with the help of fusion with MRI. CTV confirmed planning target volume (PTV). The dose of PTV occurred at the 80 % isodose, encircling the PTV. The RT procedure in group A is conducted as IMRT (tomotherapy or step-and-shoot IMRT or volumetric modulated arc therapy) 24 Gy in a single fraction at the 80 % isodose. In group B, RT is performed as irradiation of the involved vertebral body as well as the ones immediately above and below to a total dose of 30 Gy with a single daily dose of 3 Gy using 6 MV individually formed beams (Linac, multileaf collimator) after CT scan-based 3D planning. The same tolerance doses of the organs at risk as in the RTOG 0631 study are used [15].

### Therapy drop-out criteria

- At the patient’s wish
- Medical condition requiring the discontinuation of therapy in the opinion of the study director or patient
- Insufficient compliance
- Medical or ethical aspects that make it impossible to continue the study
- Participant recruiting difficulties that involve an unreasonable prolongation of the study duration
- Adverse reactions that have not yet been reported in their form, severity, duration, and impact
- Unexpectedly high incidence of already-known adverse reactions
- By official decision.

### Statistical analysis

The total number of patients undergoing RT in the Department of Radiation Oncology at the University Hospital Heidelberg for metastatic processes in the vertebral column in the recruitment period is about 120, and about 90 of them shall fulfill the inclusion criteria within 1 year. On account of the explorative character of this study, it is not possible to estimate the total number of cases; however, with a scheduled number of 30 patients per group, it will be possible to detect a standardized mean value effect of 0.74 with a power of 80 % and a significance level of 5 % when a normal distributed variable is examined. The primary endpoint is a categorical variable (the percentage of patients achieving at least a 2-point decrease in pain according to the VAS). Means, standard deviations, medians, and minimum and maximum or absolute and relative frequencies will be reported according to the scale level of the variables. The results will be reported as *P* values. For all analyses, a *P* value of 0.05 or less will be considered significant, and results of appropriate statistical tests will be of a descriptive nature and have no confirmatory value. Wherever possible, statistical graphics will illustrate the findings.

### Ethical issues, information, and safety

The study protocol, patient information sheet, and declaration of informed consent were submitted to the Heidelberg University Ethics Committee. Approval was given in 2013 (#S-431/2013). Additionally, approval was given from the federal office of radiation protection in Germany. Insurance for recruited patients was provided. The study directors shall immediately notify the Ethics Committee of any study protocol changes that may have an impact on the safety of the patients. Furthermore, the Ethics Committee shall be notified of all severe adverse events reported to the study directors and of the regular or premature termination of the study. The procedures described in the submitted study protocol regarding the performance, evaluation, and documentation of this study were selected in such a way that the principles of the Good Clinical Practice guidelines are observed. The regulations regarding medical confidentiality and data protection are fulfilled.

## Discussion

The aim of this explorative study is to evaluate in a randomized fashion the pain relief of two different RT techniques. A comparable study was recently conducted without randomization [15]. QoL, fatigue, local control, and survival are secondary endpoints. Bone metastases are a very frequent secondary diagnosis associated with advanced tumor disease, and the vertebral column is the most frequent localization [16, 17]. Spinal bone metastases are an important factor related to QoL in an advanced tumor stage. Patients affected by this condition are usually immobilized and this is due primarily to the risk of pathological fractures and the related risk of spinal cord compression. Palliative percutaneous RT is one of the most important therapeutic options available in this regard. Therapy goals are a reduction in pain and fatigue, improvement of QoL, and prevention of pathological fractions or neurological deficits. Recent trials demonstrated the feasibility and accurate use of SBRT to treat spinal metastases, with rigorous quality control, in a cooperative group setting. This technique allows the application of a high dose at the tumor region in the localized spine. The spinal cord is the dose-limiting organ at risk for SBRT. Radiation myelopathy usually occurs more than 6 months but fewer than 3 years after the end of the treatment [18]. Stereotactic radiosurgery delivers a highly conformal radiation dose to a small target in a single fraction with a sharp fall-off in the surrounding areas. The aims of this explorative study are to investigate pain relief after conventional fractionated RT and single-fraction IMRT and to evaluate local control and overall and bone survival and also to assess other clinical parameters such as pain, QoL, and fatigue as secondary endpoints. On the basis of this study, if pain relief and local control are superior in the single-dose group and there are no side effects, the results would offer a rationale for future large controlled studies as non-inferiority trials to confirm these findings.

## Trial status

This trial is currently recruiting.

## Abbreviations

CT: Computed tomography
CTV: Clinical target volume
IMRT: Intensity-modulated radiotherapy
MRI: Magnetic resonance imaging
PTV: Planning target volume
QoL: Quality of life
RT: Radiotherapy
SBRT: Stereotactic body radiation therapy
VAS: Visual analog scale
